# Validation of the Bar-On EQ-i: YV (S) Inventory in Its Spanish Version: Gender-Based Invariance Analysis

**DOI:** 10.3390/ijerph18041643

**Published:** 2021-02-09

**Authors:** Raquel Gilar-Corbi, María-Virtudes Valdés, Leandro Navas, Francisco Pablo Holgado-Tello, Juan-Luis Castejón

**Affiliations:** 1 Department of Developmental Psychology and Didactics, University of Alicante, 03080 Alicante, Spain; virtudes.valdes@ua.es (M.-V.V.); leandro.navas@ua.es (L.N.); jl.castejon@ua.es (J.-L.C.); 2 Department of Behavioral Sciences Methodology, Faculty of Psychology, National University of Distance Education (UNED), 28040 Madrid, Spain; pfholgado@psi.uned.es

**Keywords:** emotional intelligence, Bar-On EQ-i: YV (S), factorial structure, psychometric properties, model invariance

## Abstract

The purpose of this work is to verify the factorial structure and analyze the reliability of the Emotional Quotient Inventory (EQ-i): Youth Version (YV) (S) by evaluating emotional intelligence in a more extensive sample of Spanish adolescents than has been used to date, since this inventory has been employed in various studies but with a very limited number of participants. For this study, 5292 adolescents from all over Spain participated—male (51.2%) and female (48.8%) secondary education students between 11 and 19 years old, with an average age of 14.33. Data analysis included a Confirmatory Factor Analysis (CFA), reliability analysis, and model invariance as a function of gender. The CFA confirms that the data empirically support the theoretical model and that the goodness-of-fit indexes are adequate. The reliability analysis of the inventory presents a Cronbach’s alpha coefficient for the total scale of 0.76, and reliability indexes for each of the factors range between 0.63 and 0.80. The findings show that the model indicates invariance related to gender.

## 1. Introduction

New works on intelligence address the affective side of individuals [1]. Thorndike [2] first introduced the importance of the social part of intelligence and used the term “social intelligence” to describe the ability to comprehend and understand other people and act in human relationships. From this type of intelligence derives the social competence and social skills that are currently known as emotional intelligence (EI) [3]. Gardner [4,5] raised a notion of intelligence that embraces different cultures, introducing the concept of “multiple intelligences” and highlighted capacities that had not been previously taken into account by considering up to eight independent types of intelligence, among which are included interpersonal and intrapersonal intelligence (related to factors included in some EI theories). These latter types of intelligence contribute to the emergence of the term “emotional intelligence” (EI), coined by Salovey and Mayer [6], who defined it as a construct formed by four components: perceiving, using emotions in order to facilitate thought, understanding, and managing emotion.

This idea was published in 1995 in the work of Goleman [7], who defined emotional intelligence as a person’s ability to recognize feelings in oneself and in others. Currently, it has been suggested that IQ alone does not guarantee success in life; other abilities give us a sense of well-being and can predict, in part, one’s achievement [7]. This author highlights up to five components of EI: Motivation, Self-Regulation, Empathy, Self-Awareness, and Social Skills.

In the attempt to define the concept of EI, two main models can be extracted from the existing literature: (a) Ability models focus on the processing of emotional information and the skills related to this processing. Mayer and Salovey [8] framed their theory on EI in this model, analyzing the concept of emotional intelligence as a mental ability [9]. (b) In the trait EI perspective, emotional skills are combined with aspects of personality. This perspective focuses on the emotional aspects of personality (emotion-related dispositions). Petrides [10] formulates his theory on EI from this perspective. The Bar-On Theory [11] is also close to this mixed approach. It is important to mention that these models are not totally mutually exclusive [12,13,14].

According to the Bar-On model [11], EI includes a set of competencies, skills, and facilitators. This multifactorial set is the one that will allow the person to understand himself, understand others, relate effectively with them, and be able to respond to the situations that he faces daily. Thus, the Bar-On model comprises 10 key components: Self-Regard, Interpersonal Relationships, Impulse Control, Problem-Solving, Emotional Self-Awareness, Flexibility, Reality-Testing, Stress Tolerance, Assertiveness, and Empathy. The model also includes five facilitators: Optimism, Self-Actualization, Happiness, Independence, and Social Responsibility.

The importance of emotional intelligence has been demonstrated. Different studies have shown a relationship between EI and psychological adjustment, better satisfaction with life in general, and good social or academic adaptation [15,16,17,18,19,20,21,22,23,24], verifying emotional intelligence’s positive effect on a person’s psychological well-being. It has also been proven that emotional intelligence is related to disruptive behaviors, such that children with low emotional intelligence indexes display a greater number of socially inappropriate behaviors [17,24,25,26,27,28]. Moreover, emotional intelligence and academic performance have been shown to be related, with researchers finding significant correlations between both variables [27,29,30,31,32,33,34]. Similarly, some studies associate emotional intelligence with addictive behaviors, with low emotional intelligence being a prominent predictor in the addictive type of disorders potentially developed by subjects [35,36,37,38,39,40,41,42]. Over time, different instruments have been created to assess individuals’ level of EI. At first, the research focused on the study of a fairly young population, but this focus has been modified, since this stage is always changing; thus, the study age has been extended, which allows more stable conclusions to be drawn [43].

There are, therefore, different tests that aim to assess emotional intelligence: mixed models and ability-based models [44] predominate. Mixed models attempt to establish a relationship between personality and emotional intelligence. These models are based on personality traits and implement the instruments created by authors such as Bar-On [45], Oriolo and Cooper [46], Boyatzis, Coleman and Rhee [47], Mestre [48], Goleman [7], and Petrides [49]. Ability-based models focus on how emotional intelligence is used in the processing of information and in the study of the capacities that are related to such processing [6,50,51].

An inventory widely used in our context to assess emotional intelligence is the Bar-On Emotional Quotient Inventory (EQ-i) [45], which identifies the degree to which the emotional components are presented. This inventory comprises five components (Intrapersonal, Interpersonal, Stress Management, Adaptability, and General Mood) that are broken down into a total of 15 subscales and encompass 133 items that are answered by a five-point Likert scale (from very seldom true to very often true); additionally, items that measure the number of random responses or the distortion of the participants’ responses are added to ensure social appropriateness. This inventory has a version of 60 items and a shorter version of 30 items and has been translated into several languages, including Spanish. This inventory is the basis of the version for children and adolescents, intended for people from 7 to 18 years old, called the Bar-On EQ-i: YV (S) inventory by Baron and Parker [52], and translated into Spanish by Caraballo and Villegas [53].

We have selected The Bar-On Emotional Quotient Inventory: Youth Version (EQ-i: YV) for our research because this instrument has shown that its evaluation of EI is related to physical and psychological health [54,55], social and academic adaptation [56], academic performance [57], and a lower risk of substance use [58]. All of these are relevant to the educational stage on which we have focused our study.

In Spain, Ferrándiz et al. [59] used the version comprising 60 items, excluding the Positive Impression scale, and validated it using an Exploratory Factor Analysis (EFA) in a sample of 1655 students from the Murcia region aged between 6 and 18 years, obtaining a five-factor structure (Mood, Stress Management, Adaptability, Interpersonal, and Intrapersonal) comprising items with factorial loads similar to the original and reliability scores for dimensions between 0.63 and 0.80. In the validation by López-Zafra, Pulido, and Berrios-Martos [60], a short version of 30 items was used, eliminating the Positive Impression scale, with a sample of 390 university students from Granada, Jaén, and Murcia, aged between 18 and 32 years. These authors reduced the number of items to 28 after performing EFA and Confirmatory Factor Analysis (CFA), grouping them into four factors (Interpersonal, Intrapersonal, Stress Management, and Adaptability) that present an internal consistency ranging between 0.70 and 0.78. In the study by Esnaola et al. [61], the structure of the short 30-item inventory was validated, without considering the Positive Impression scale, using a sample of 508 students from the Basque region aged between 11 and 19 years old. After conducting EFA and CFA, they obtained four first-order factors (Interpersonal, Intrapersonal, Stress Management, and Adaptability) and one second-order factor (Emotional-Social Intelligence), with an internal consistency that ranges between 0.67 and 0.83. Robles-Bello et al. [62] analyzed psychometric properties of the Bar-On Emotional Quotient Inventory: Youth Version (EQ-i: YV) in adults with Down syndrome, employing an exploratory and a confirmatory factor analyses, confirming the five-factor structure of the EQ-i: YV. Sánchez-Teruel et al. [63], validated and analyzed the psychometric properties of the EQ-i: YV scale in Spanish adults with Down syndrome, and they proposed a new version of the scale, EQ-i: SVDS, comprising four factors: Mood, Stress Management, Interpersonal, and Intrapersonal. Robles-Bello and Sánchez-Teruel [64] validated the Emotional Quotient Inventory: Youth Version (EQ-i: YV) in adolescents with Down syndrome, confirming that the instrument is valid for measuring EI in these populations.

There are studies in which girls obtained higher average scores on the Interpersonal scale [65,66]; in contrast, no significant differences were established between sexes in the total values of Emotional Intelligence [67,68,69]. In other investigations, however, women scored highest on the Emotional Intelligence index [51,66,70,71,72]. Therefore, it is worth looking into whether the model varies by gender.

The objectives of this study were to verify the factorial structure proposed by the authors of the EQ-i: YV (S), analyze its reliability, and verify model invariance by gender and with respect to age in a much broader sample composed of adolescents from the entire peninsula and the Balearic archipelago (16 autonomous communities in total). The originality of this work lies precisely in these two aspects: involving adolescents from virtually all of Spain and assessing model invariance in terms of gender, which has been scarcely studied.

## 2. Materials and Methods

### 2.1. Participants

A total of 5385 secondary education students (compulsory education obligatory and post-compulsory education obligatory) participated, and 93 of them were discarded for not providing informed consent or for not responding to demographic data or any of the elements of the scales; thus, a total of 5292 adolescent secondary education students (51.2% boys; 48.8% girls) aged between 11 and 19 (Mean = 14.33 ± 0.02, Standard Deviation = 1.65) participated in the study. Sixty-two participants, 1.2%, were 11 years old; 748, 14.1%, were 12; 999, 18.9%, were 13; 1121, 21.2%, were 14; 1086, 20.5%, were 15; 666, 12.6%, were 16; 486, 9.2%, were 17; 90, 1.7%, were 18; and 34, 0.6%, were 19. They came from 33 public (45.5%) and privately subsidized (54.5%) educational centers of the country’s different autonomous communities (except for the Canary Islands, Ceuta, and Melilla). Table 1 shows the distribution of participants by autonomous communities. Of all the participants, 19.2% were enrolled in the first year of compulsory secondary education (ESO), 19.6% in the second year, 23.2% in the third year, and 19.7% in the fourth year; 8% were enrolled in the first year of high school (Bachillerato), and 6.9% in the second year; 1.5% were enrolled in the first year of vocational training (Formación Profesional, FP), and 1.7% in the second year. Regarding the sample’s ethnic composition, most of the students belonged to the Mediterranean ethnic group, although 4.42% were Romani, 7.30% were Hispanic Americans, and 6.12% were of Maghrebian and Sub-Saharan African origin.

### 2.2. Materials

The Emotional Intelligence Inventory: Young Version (Short) by Bar-On and Parker [52] was used in its Spanish translation by Caraballo and Villegas [53]. The instrument included 30 items with a 4-point Likert scale (1 represents not true in my case, 2 a little true in my case, 3 true in my case, and 4 very true in my case). The scoring scale of Items 5, 8, 9, 12, 16, 26, 27, and 29 is reversed. An error was detected for one reversed item in the Spanish translation—in the correction of the 60-item version, Item 35 is reversed and corresponds to item 17 in the short version (“I get angry easily”), but the correction template does not indicate that it should be reversed, and, conversely, the scale is reverse-scored in Item 16, and it should not be. The scale is composed of four factors: the A factor, the Intrapersonal scale, is composed of Items 2, 6, 12, 14, 21, and 26 and assesses one’s ability to understand an individual’s emotions and to convey those emotions to others; the B factor, the Interpersonal scale, which includes Items 1, 4, 18, 23, 28, and 30, refers to one’s ability to have adequate and satisfactory interpersonal relationships and to understand, consider, or appreciate the emotions of others; the C factor, the Stress Management scale, groups Items 5, 8, 9, 17, 27, and 29 and assesses the ability to manage and control an individual’s emotions and to respond calmly to stressful events; the D factor, the Adaptability scale, is composed of Items 10, 13, 16, 19, 22, and 24 and refers to one’s ability to be flexible, realistic, and effective in solving problems and managing changes. A value is established for the total scale, the Total EQ (E factor), which is calculated by summing up the scores of the four previous subscales. The F factor, the Positive Impression scale, comprises Items 3, 7, 11, 15, 20, and 25 and measures the number of random answers or responses marked by the participants to ensure that the inventory will be socially appropriate. The internal consistency of each scale varies between 0.50 and 0.77, depending on sex and age [66].

### 2.3. Procedure

First, approval was requested and obtained by the Ethics Committee of the competent authority (Ref. UA-2017-01-11). Subsequently, a list of public and private centers of each autonomous community was obtained, and a random selection was contacted until we had a large sample and a representation of different communities of Spain. The researchers proceeded to directly contact, in person, by phone, by mail, or by email, the Department Heads, Programme Directors, and Councillors of the various educational centers to request their collaboration in the investigation, describing the purpose of the study and its voluntary, confidential, and anonymous nature. The inventory was also added so that it could be reviewed by the administration. Afterwards, the non-compensated informed consent of the families was obtained for participants who were minors. Finally, we proceeded to have students complete the inventory, in class time. The students followed the instructions that had been previously provided to the administering teachers, which took between 10 and 15 min.

### 2.4. Design and Data Analisys

This study uses an ex post facto descriptive design. The data obtained were subject to factor analysis, reliability analysis, and model invariance by gender. To carry out the analyses, the statistical program packages SPSS version 20 (IBM Corporation, Armonk, NY, USA) and LISREL version 8.80 (Scientific Software International, Chicago, IL, USA) were used. Based on the parameters proposed by the authors, explained in the section that describes how the instrument is used, a CFA was carried out employing the Robust Unweighted Least Squares (RULS) estimation method [73].

## 3. Results

### 3.1. Descriptive Statistics

Table 2 shows the descriptive analysis of item scores. Most of the skewness and kurtosis indices indicate deviations from the normal curve.

### 3.2. Confirmatory Factor Analysis (CFA)

**Figure 1:**
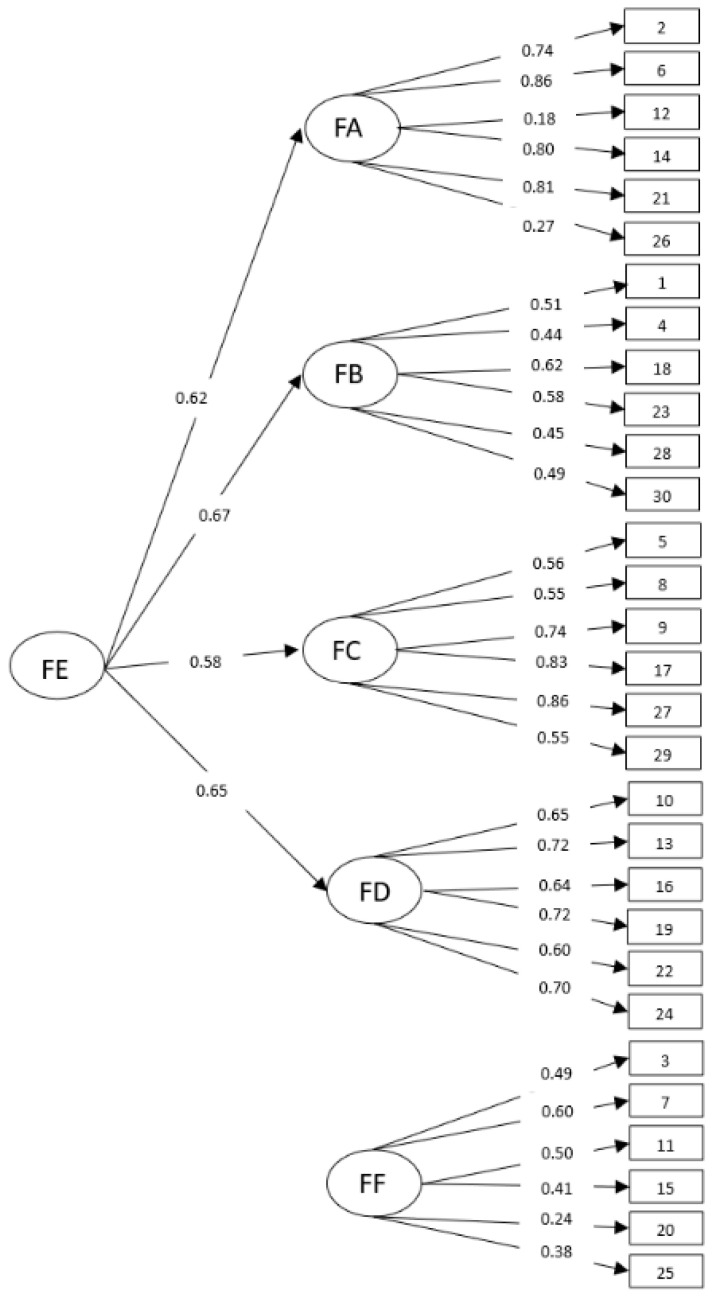
Confirmatory Factor Analysis (CFA) final model of the Spanish version of the Bar-On Emotional Quotient Inventory: (EQ-i): Youth Version (YV) (S). Note: FA = Intrapersonal scale; FB = Interpersonal scale; FC = Stress Management scale; FD = Adaptability scale; FE = EI Total scale; FF = Positive impression scale.

Due to the ordinal nature of the items, a matrix of polychoric correlations was used, as is recommended for ordinal variables [74,75,76,77,78]. The goodness-of-fit indexes obtained were as follows: χ^2^ = 3620.30 (*p* = 0.00); Root Mean Square Error of Approximation (RMSEA) = 0.05 (90% confidence interval: 0.04 and 0.05); Comparative Fit Index (CFI) = 0.95; Root Mean Square Residual (RMR) = 0.06; Standardized Root Mean Square Residual (SRMR) = 0.06; Goodness-of-Fit Index (GFI) = 0.94; Adjusted Goodness-of-Fit Index (AGFI) = 0.93. Figure 1 shows the completely standardized solution for the model. As these results show, the theoretical model is empirically supported by the data.

The bivariate correlation analyses using the Pearson r coefficient indicate that the five factors have positive and statistically significant relationships with the total EQ-i: with the Intrapersonal scale (r = 0.59; *p* = 0.00), with the Interpersonal scale (r = 0.56; *p* = 0.00), with the Stress Management scale (r = 0.49; *p* = 0.00), and with the Adaptability scale (r = 0.63; *p* = 0.00). Table 3 shows the correlation matrix.

### 3.3. Internal Reliabilies

For the total Bar-On EQ-i: YV (S), considering the Positive Impression scale, Cronbach’s alpha (α) and McDonal’s Omega (Ω) are 0.76. When this scale is omitted, the value of α is 0.73, which would increase to 0.74 if Item 12 were eliminated; the value of Ω is 0.747, which would increase to 0.751 if Item 12 were eliminated. For the Intrapersonal scale, α was 0.74, and Ω was 0.77; for the Interpersonal scale, α = 0.63 and Ω = 0.63. For the Stress Management scale, α = 0.79 and Ω = 0.80, and the Adaptability scale shows α and Ω values of 0.80. The internal consistency of the Positive Impression scale is α = 0.50 and Ω = 0.52.

### 3.4. Model Invariance by the Variable Gender and Differences with Respect to Age

For the gender variable, factor structure validity was tested employing multi-group analysis. Afterwards, invariance of factor loadings, gamma parameters, and factor error variances/co-variances of the first order factors were examined.

To determine the differences with respect to age, the following grouping was taken into consideration: participants aged 11–12 years, 13–14, 15–16, and 17–19. An ANOVA of the different dimensions of the Bar-On EQ-i: YV (S) was carried out. The results indicate that the differences with respect to age are statistically significant in the Stress Management, Adaptability, Total EQ, and Positive Impression scales, for which the post hoc test is applied in order to determine between which groups these differences existed. No differences were registered regarding sex in the Interpersonal and Intrapersonal scales, unlike the study by Sanmartín, Gonzálvez, and Vicent [79], who found differences in the interpersonal scale. Adolescents between 11 and 12 obtained higher mean scores in our study on the Stress Management, Total EQ, and Positive Impression scales. The latter indicates that younger students are more likely to answer what is considered socially appropriate and to perceive themselves with a higher level of emotional intelligence at a general level. However, the scores obtained in this factor in all age ranges are low, which indicates that in general the participants are not responding based on their social desirability. There are relevant studies that relate emotional intelligence to age [80]; in our study, older participants showed higher levels of emotional intelligence.

### 3.5. Baseline Model

Before analyzing gender invariance through a multigroup analysis, the model was tested in each gender group separately.

The global fit indexes obtained regarding boys were χ^2^ = 2759.18 (*p* = 0.00; *df* = 391), GFI = 0.93; AGFI = 0.91; CFI = 0.94, and RMSEA = 0.05. Regarding girls, the global fit indexes were χ^2^ = 2044.07 (*p* = 0.00; *df* = 391), GFI = 0.94; AGFI = 0.93; CFI = 0.96, and RMSEA = 0.04. The standardized solutions for both genders are presented in Table 4.

### 3.6. Testing the Validity of the Model

Once we have empirical evidence about the fit of the model in both groups separately, in order to test if the model is suitable taking into account both groups simultaneously, we estimated all the parameters in a multigroup analysis without any restrictions about the equivalence between parameters.

The goodness-of-fit indices were as follows: χ^2^ = 5141.66 (*p* = 0.00; *df* = 782), GFI = 0.94; CFI = 0.95; ECVI = 1.09; RMSEA = 0.05.

The global fit indices (Table 5) for Model 1 are adequate, that is, it could be reasonably concluded that the model represents how the data are related. Nevertheless, it is necessary to explore if the loading across gender is invariant.

### 3.7. Testing the Invariance of Factor Loading across Gender

To examine the invariance of factor loading, we conducted a multigroup analysis imposing equality constraints on the factor loadings of the items. Firstly, all factor loadings were constrained to be equal across gender (Model 2).

The global fit indexes of Model 2 (Table 5) supported the hypothesis of invariant factor loadings. The increase in χ^2^ in comparison to the baseline model (Model 1) was not significant across gender groups (∆χ^2^ = 0.94; ∆*df* = 25). Consequently, the factor structure was shown to be equivalent across groups, and the measurement model was shown to be defined by the factor loadings of the items.

### 3.8. Testing the Invariance of Gamma Parameters

Next, we focused attention on the structural model. Firstly, we examined the equivalence in the relationships between FE and the first-order factors (FA, FB, FC, and FD) [81,82]. Multi-group analyses were performed imposing equality constraints on the gamma parameters. That is, all gamma parameters were constrained to be equal across gender (Model 3).

Again, the global fit indexes supported the hypothesis of equivalence in these parameters of the structural model (Table 5). The increment in χ^2^ of Model 3 in comparison to the baseline model (Model 1) was not significant across gender groups (Λχ^2^ = 6.13; Λ*df* = 29). Therefore, the gamma parameters may be considered equivalent across groups.

### 3.9. Testing the Invariance of the Error of the First Order Factors

The analysis was carried out in different phases: firstly, all factor error variances/co-variances were constrained to be equal across gender (Model 4); secondly, only the factor error variances (Model 5) were constrained; thirdly, only the factor error co-variances were constrained (Model 6); afterwards, each individual factor variance and each individual factor covariance were constrained.

In terms of gender, only two error variances are invariant: the error of Factor 1 (intrapersonal scale) and the error of Factor 3 (Stress Management scale). Table 5 shows the rest of the error variance, and the covariances are variant between gender groups.

## 4. Discussion

The first objective of the research was to replicate the factorial structure of the EQ-i: YV (S) Inventory by Baron and Parker [52] in its translation into Spanish [53] with a large number of secondary education students of all the autonomous communities, except for the Canary Islands and the autonomous cities of Ceuta and Melilla. The results obtained in the CFA confirm the existence of the different subscales that comprise the inventory, and the completely standardized solution for the model indicates empirical support for the model, which is in line with the results of the works of Ferrándiz et al. [59], López-Zafra et al. [60], and Esnaola et al. [61]. These results confirm the Intrapersonal, Interpersonal, Stress Management and Adaptability scales as dimensions that make up the Emotional Intelligence Index, in addition to the Positive Impression dimension. In addition, the factors obtained show statistically significant correlation coefficients between them, and the internal consistency indexes range between 0.63 and 0.80 (when excluding the Positive Impression scale), values that are within the required range in terms of the reliability of psychological tests; that is, they are adequate, as confirmed in previous research [59,61,83].

A number of extensively used global fit indices for the multigroup CFA models were examined. The results of the invariance of the factor model could suggest that the model is reasonably invariant across gender groups. That is, a five-factor structure was appropriate across gender; second, the factor loading was shown to be invariant across gender groups; additionally, the relationships between FE and FA, FB, FC, and FE could be considered as equivalent for gender, which would support the results of other studies [67,68,84].

No differences were registered on the Interpersonal and Intrapersonal scales. Adolescents between 11 and 12 years old obtained higher mean scores on the Stress Management, Positive Impression, and Total EQ scales, which indicates that they perceive themselves with a higher level of emotional intelligence at a general level. There are relevant studies regarding age with specific variables such as aggressive behavior and anxiety [85], but fewer studies have related emotional intelligence with age [80], indicating that older participants—using the same inventory as this study—have shown higher levels of emotional intelligence.

Despite the fact that the construct validity study was shown to be correct and stable, given the variance in the error terms of the first order factor, more research needs to be done to deepen the analysis of gender differences.

The differences in the error variances between boys and girls may be due to factors in the model that are not considered, such as the student’s ability to reflect on himself or herself and the ability to understand the items or the level of cognitive development; these omissions could explain such differences. In this sense, it would be appropriate to inquire about which factors could generate these discrepancies. This inquiry could constitute future lines of work.

In educational data, the nested data could emerge as a relevant issue and they could be/become very useful to specified more general effects. In this sense, in order take this aspect into account in forthcoming researches, multilevel models through hierarchical linear modelling should investigated. That is, variables that represent the schools (variables of level 2 in hierarchical linear modelling) should be operationalized in order to try examining if they influence other variables related to emotional intelligence. Another important limitation that opens future research questions is related to the reliability of the Interpersonal and Positive Impression scales. Both measures are low, and this could indicate a problem of representativeness of the dimensions. Although there is empirical evidence about the internal structure of the dimensions, they are not precise at all, probably because both constructs need to be measured by a higher number of items. Thus, future research could focus on content validity, so as to find more relevant indicators of these dimensions.

Finally, these results could constitute empirical evidence of the invariance of the measurement model and of the Bar-On EQ-i: YV (S). Nevertheless, more research is needed to explain the theoretical differences across gender. Further, the invariance of the model according to the course could be analyzed.

## 5. Conclusions

Based on the obtained global fit indices, the factor pattern coefficients were statistically invariant, like the structural model regarding gamma parameters. However, for factor error variances/co-variances, the invariance was uncertain.

Given that the inventory is reliable, and the dimensional structure is confirmed, as demonstrated in the results of this study, it is possible for an instrument to assess emotional competencies based on four factors, and, in the academic context, it is important to know the emotional components that will provide adolescents with a greater capacity to adapt to their environment. This knowledge is especially relevant for various reasons.

First, emotional intelligence acts as a protective factor in regard to addictive behaviors or other types of disorders (substance addictions, behavioral-type addictions, or eating disorders). People who lack emotional abilities resort to the use of substances to reduce negative emotional states or to seek, through consumption, the pleasurable emotional states that they lack [20,35,36,37,38].

Second, emotional intelligence plays a decisive role in psychological adjustment, as demonstrated by the fact that people with greater emotional capacity are those who present with less depression and anxiety, which makes them more optimistic and more psychologically adjusted and leads to more age-appropriate behaviors, fewer disruptive behaviors, and better academic results [16,17,18,23,24,30,31,32,33,34,40,41,42,49].

Third, in educational centers, there is growing concern about coexistence and bullying. It has been proven that both the aggressor and the victim have emotional intelligence deficiencies: the victim lacks strategies to cope with the harassment he or she is receiving, and the aggressor has poor self-control, lacks empathy and the ability to perceive the pain he or she is causing, and lacks prosocial behaviors and abilities that are a part of emotional intelligence [19,25,26].

The findings therefore indicate that further research on emotional intelligence in adolescents is necessary, because good levels of emotional intelligence are related to addictions, psychological adjustment, and coexistence in general.

## Figures and Tables

**Table 1:** Frequency distribution of participants by autonomous community.

Community	*f_a_*	*f_r_*
Cataluña	279	5.3
C.Valenciana	1740	32.9
Asturias	249	4.7
Castilla y León	207	3.9
Murcia	227	4.3
Castilla La Mancha	867	16.4
Andalucía	462	8.7
Madrid	310	5.9
Galicia	105	2.0
Baleares	84	1.6
La Rioja	144	2.7
Navarra	106	2.0
País Vasco	93	1.8
Cantabria	232	4.4
Extremadura	87	1.6
Aragón	100	1.9
Total	5292	100.0

Note: *f_a_* = absolute frequency; *f_r_* = relative frequency.

**Table 2:** Descriptive statistics for items scores.

Items	Mean	Std. Deviation	Skewness	Kurtosis
B1	2.91	0.89	−0.39	−0.67
B2	2.02	0.91	0.58	−0.46
B3	2.01	0.94	0.54	−0.70
B4	3.44	0.71	−1.12	0.83
B5	1.92	0.93	0.56	−0.81
B6	1.97	0.92	0.66	−0.43
B7	2.34	0.89	0.12	−0.74
B8	3.45	0.80	−1.41	1.31
B9	2.86	1.02	−0.51	−0.86
B10	2.67	0.86	−0.12	−0.65
B11	1.82	0.85	0.79	−0.10
B12	2.48	1.15	−0.05	−1.43
B13	2.62	0.86	−0.04	−0.67
B14	2.01	0.95	0.60	−0.60
B15	3.15	0.82	−0.63	−0.34
B16	2.84	0.86	−0.23	−0.69
B17	2.84	0.94	−0.50	−0.62
B18	2.92	0.84	−0.31	−0.63
B19	2.67	0.83	−0.06	−0.60
B20	1.47	0.77	1.70	2.35
B21	1.92	0.93	0.75	−0.36
B22	2.68	0.89	−0.11	−0.77
B23	2.98	0.90	−0.42	−0.77
B24	2.57	0.87	0.00	−0.68
B25	1.61	0.86	1.32	0.86
B26	2.89	1.02	−0.59	−0.76
B27	2.85	1.02	−0.49	−0.89
B28	2.90	1.02	−0.50	−0.91
B29	2.40	1.06	0.03	−1.25
B30	3.03	0.91	−0.55	−0.67

**Table 3:** Correlation matrix.

	FA	FB	FC	FD	FF	FE
FA	-	0.11 **	0.05 **	0.15 **	0.24 **	0.59 **
FB	0.11	-	−0.04 **	0.36 **	0.17 **	0.56 **
FC	0.05 **	−0.04 **	-	−0.03 *	0.12 **	0.49 **
FD	0.15 **	0.36 **	−0.03 *	-	0.27 **	0.63 **
FF	0.24 **	0.17 **	0.12 **	0.27 **	-	0.33 **
FE	0.59 **	0.56 **	0.49 **	0.63 **	0.33 **	-

Note: FA = Intrapersonal scale; FB = Interpersonal scale; FC = Stress Management scale; FD = Adaptability scale; FE = EI Total scale; FF = Positive impression scale. ** *p* ≤ 0.01; * *p* ≤ 0.05.

**Table 4:** Standardized solutions of the model for males and females. The last line shows the gamma parameters.

Item	Intrapersonal Males Females		Interpersonal Males Females		Management Males Females		Adaptability Males Females		Impression Males Females	
B1	---	---	0.49	0.51	---	---	---	---	---	---
B2	0.75	0.73	---	---	---	---	---	---	---	---
B3	---	---	---	---	---	---	---	---	0.47	0.49
B4	---	---	0.46	0.44	---	---	---	---	---	---
B5	---	---	---	---	0.60	0.52	---	---	---	---
B6	0.88	0.83	---	---	---	---	---	---	---	---
B7	---	---	---	---	---	---	---	---	0.59	0.61
B8	---	---	---	---	0.56	0.55	---	---	---	---
B9	---	---	---	---	0.75	0.73	---	---	---	---
B10	---	---	---	---	---	---	0.66	0.63	---	---
B11	---	---	---	---	---	---	---	---	0.46	0.52
B12	0.21	0.14	---	---	---	---	---	---	---	---
B13	---	---	---	---	---	---	0.72	0.72	---	---
B14	0.81	0.80	---	---	---	---	---	---	---	---
B15	---	---	---	---	---	---	---	---	0.42	0.43
B16	---	---	---	---	---	---	0.64	0.65	---	---
B17	---	---	---	---	0.84	0.81	---	---	---	---
B18	---	---	0.58	0.65	---	---	---	---	---	---
B19	---	---	---	---	---	---	0.71	0.72	---	---
B20	---	---	---	---	---	---	---	---	0.24	0.20
B21	0.82	0.79	---	---	---	---	---	---	---	---
B22	---	---	---	---	---	---	0.62	0.59	---	---
B23	---	---	0.57	0.58	---	---	---	---	---	---
B24	---	---	---	---	---	---	0.69	0.71	---	---
B25	---	---	---	---	---	---	---	---	0.43	0.31
B26	0.33	0.22	---	---	---	---	---	---	---	---
B27	---	---	---	---	0.87	0.85	---	---	---	---
B28	---	---	0.45	0.41	---	---	---	---	---	---
B29	---	---	---	---	0.56	0.53	---	---	---	---
B30	---	---	0.51	0.44	---	---	---	---	---	---
EI total	0.69	0.70	0.78	0.78	0.66	0.66	0.73	0.73	---	---

**Table 5:** Invariance by gender.

Model	χ^2^ (Λχ^2^)	*df* (Λ*df*)	ECVI	RMSEA	GFI	CFI	NFI	TLI
Number of factors invariant (Model 1)	5141.66	782	1.09	0.04	0.94	0.95	0.94	0.94
Pattern of factor loadings (Model 2)	(−0.94)	(25)	1.08	0.04	0.94	0.94	0.94	0.94
GA_IN (Model 3)	(−6.13)	(29)	1.08	0.04	0.94	0.94	0.94	0.94
PS_IN (Model 4)	(164.96) *	(44)	1.10	0.04	0.94	0.95	0.94	0.94
VAR_IN (Model 5)	(70.12) *	(34)	1.09	0.04	0.94	0.95	0.94	0.94
Ps11_IN	(30.24)	(30)	1.08	0.04	0.94	0.95	0.94	0.94
Ps22_IN	(135.81) *	(31)	1.10	0.04	0.94	0.95	0.94	0.94
Ps33_in	(−40.76)	(31)	1.07	0.04	0.94	0.95	0.94	0.94
Ps44_in	(151.25) *	(32)	1.11	0.04	0.94	0.95	0.94	0.94
Ps55_in	(165.47) *	(32)	1.11	0.04	0.94	0.95	0.94	0.94
COVA_IN(model6)	(178.67) *	(41)	1.11	0.04	0.94	0.95	0.94	0.94
Cov_12_in	(218.75) *	(32)	1.12	0.04	0.94	0.95	0.94	0.94
Cov_13_in	(144.41) *	(32)	1.10	0.04	0.94	0.95	0.94	0.94
Cov_14_in	(84.63) *	(32)	1.09	0.04	0.94	0.95	0.94	0.94
Cov_15_in	(146.33) *	(32)	1.10	0.04	0.94	0.95	0.94	0.94
Cov_23_in	(190.4) *	(32)	1.11	0.04	0.94	0.95	0.94	0.94
Cov_24_in	(218.39) *	(32)	1.12	0.04	0.94	0.95	0.94	0.94
Cov_25_in	(68.08) *	(32)	1.09	0.04	0.94	0.95	0.94	0.94
Cov_34_in	(53.94) *	(32)	1.09	0.04	0.94	0.95	0.94	0.94
Cov_35_in	(97.68) *	(32)	1.09	0.04	0.94	0.95	0.94	0.94
Cov_45_in	(156.04) *	(32)	1.11	0.04	0.94	0.95	0.94	0.94

Note: ∆χ^2^ (the increase of the χ^2^ statistic relative to the baseline model due to the additional invariance constraint(s) on parameters) and ∆*df* (*df*: difference between the two models). * *p* < 0.05.

## Data Availability

Data available on request from the authors.
